# Structured nursing follow-up: does it help in diabetes care?

**DOI:** 10.1186/2045-4015-3-27

**Published:** 2014-08-29

**Authors:** Michal Shani, Sasson Nakar, Alex Lustman, Amnon Lahad, Shlomo Vinker

**Affiliations:** 1 Department of Family Medicine Central District, Clalit Health Service, Rehovot, Israel; 2 Department of Family Medicine Sackler Faculty of Medicine, Tel Aviv University, Tel Aviv, Israel; 3 Family Medicine Department Hadassah Medical School, The Hebrew University, Jerusalem, Israel

**Keywords:** Diabetes mellitus, Family physicians, Nurses, Primary care

## Abstract

**Background:**

In 1995 Clalit Health Services introduced a structured follow-up schedule, by primary care nurses, of diabetic patients. This was supplementary care, given in addition to the family physician’s follow-up care. This article aims to describe the performance of diabetes follow-up and diabetes control in patients with additional structured nursing follow-up care, compared to those patients followed only by their family physician.

**Methods:**

We randomly selected 2,024 type 2 diabetic subjects aged 40–76 years. For each calendar year, from 2005–2007, patients who were “under physician follow-up only” were compared to those who received additional structured nursing follow-up care.

**Main outcomes:**

Complete diabetes follow-up parameters including: HbA1c, LDL cholesterol, microalbumin, blood pressure measurements and fundus examination.

**Results:**

The average age of study participants was 60.7 years, 52% were females and 38% were from low socioeconomic status (SES).

In 2005, 39.5% of the diabetic patients received structured nursing follow-up, and the comparable figures for 2006 and 2007 were 42.1% 49.6%, respectively. The intervention subjects tended to be older, from lower SES, suffered from more chronic diseases and visited their family physician more frequently than the control patients. Patients in the study group were more likely to perform a complete diabetes follow-up plan: 52.8% vs. 21.5% (2005; p < 0.001) 55.5% vs. 30.3% (2006; p < 0.001), 52.3% vs. 35.7% (2007; p < 0.001). LDL cholesterol levels were lower in the study group only in 2005: 103.7 vs. 110.0 p < 0.001.

**Conclusion:**

Subjects with supplementary structured nursing follow-up care were more likely to perform complete diabetes follow-up protocol. Our results reinforce the importance of teamwork in diabetic care. Further study is required to identify strategies for channeling the use of the limited resources to the patients who stand to benefit the most.

## Background

The United Kingdom Prospective Diabetes Study (UKPDS) demonstrated that improved glycemic control can prevent late complications of diabetes [1]. Prevention and early intervention of both microvascular and macro-vascular complications of Type 2 Diabetes Mellitus (T2DM) are important goals in diabetes care. Health care professionals invest time and effort in order to achieve glycemic control, and improve cardiovascular risk factors, reducing complications of diabetes.

Over the past two decades various protocols for health care delivery to diabetic patients have been suggested, so as to better utilize the professional skills of the healthcare team and improve the overall care of diabetic patients. A systematic review in 2001 found that structured recall or regular nurse contact, in addition to routine care, could bring about improved care for patients with T2DM [2].

In a previous study, patients who received automated calls with telephone nurse follow up, in addition to standard care, were more likely to perform annual cholesterol tests and poorly controlled diabetic patients showed an improvement in glycemic control compared to the control group [3]. Diabetic patients who were monitored by a nurse case manager, under the direction of a family physician, or an endocrinologist, had improved glycemic control within 12 months [4] and improved blood pressure, cholesterol and glycemic control at one year [5].

According to a Cochrane review, nurse case managers may improve patients’ diabetic control over short periods of time, but the effects in the long term are not evident [6]. Additional nurse follow-up along with structured case management, in addition to regular care, appear to improve the process of diabetic follow-up, as well as improving diabetic control at one year, but little evidence exists as to the long term effectiveness of these programs.

Since 1995 a structured follow-up protocol for diabetic patients has been performed by nurses in primary care clinics of “Clalit Health Services” (CHS), the largest health maintenance organization (HMO) in Israel. This nurse follow-up is in addition to the family physician’s regular follow-up [7]. The nurse visits include weight and blood pressure (BP) measurement, foot examination, referral for blood tests and fundus examination. In these visits, patients’ adherence to medical treatment is reviewed. Diet, exercise and foot care are discussed with the patients. The nursing follow up is scheduled 2–4 times annually. Patients are seen by the nurse or referred to the nurse by the family physician, and the nursing follow-up is performed whilst maintaining both the doctor and nurse care. The follow up appointments were scheduled for all diabetic patients in this study, even though in real life this is impractical.

Despite the time, effort and resources that have been invested in providing supplemental nursing follow-up of diabetic patients, characteristics of the selected patients and follow up efficacies have not been evaluated.

Our aim was to describe the characteristics of the patients, and to compare performance of diabetes follow-up (glucose, cholesterol and BP control) in adult diabetic patients, between those with additional structured nursing care and those monitored only by their family physician.

## Methods

The study was approved by the Hadassah Medical Organization’s ethics committee.

### Study design

A community based cohort study was performed in the Central District of Clalit Health Services (CHS) which serves over 500,000 patients. There were 29,854 diabetic patients listed on the CHS Central District register of chronic diseases during the study period.

### Setting

Israel has mandatory health insurance, provided to all citizens and permanent residents by four HMOs. CHS is the largest HMO in Israel. It serves 54% of the population and more than 70% of diabetic patients. Patient records in CHS have been completely computerized for over a decade and an extensive healthcare database has been created. The demographic data is updated directly from the population registry of the Ministry of Interior. All laboratory tests are free of charge and sent to a central lab. The lab results are reported directly to the primary care physician, and to the patient’s electronic medical file. The diagnosis of “diabetes mellitus” in the chronic disease register is estimated to be over 90% accurate [8]. The register is built by integrating information from patient files, hospital discharges, medication use and lab results.

CHS uses a passive capitation system where every patient is linked to one primary care physician. Every primary care physician is responsible for diabetes follow up for all patients on their list, including any patients seen in specialist diabetic clinics. Primary care clinics have both physicians and nurses and each has different responsibilities and tasks related to patient care. They work independently, in a coordinated manner, with the ultimate responsibility for care, being the physician’s.

### Patients

All Type 2 diabetic patients aged 40–76 years who were insured by CHS during the study period and who survived to the end of 2007, were considered eligible for the study. 2,024 diabetic patients were randomly selected from the eligible patients. Random selection of patients was done by using the control digit of the patient identity number which itself is assigned randomly.

The selected subjects were divided into two groups: patients who had physician follow-up only (control group) and patients who received additional structured nursing follow-up (intervention group). Specified separate groups were defined for the three consecutive years (2005–2007); we also compared patients who received structured nursing follow-up in all three study years to those who didn’t complete a 3 year nursing follow-up protocol.

Structured nursing follow-up is performed by the clinic nurses, in all primary care clinics, as part of routine care. The patient is scheduled to meet the nurse 2–4 times a year for 20–30 minutes per visit. The nurses, in consultation with the physicians, manage the patient list and decide who to schedule for a follow-up appointment, according to the patient’s needs and medical priorities. The follow-up includes a computerized check list of tasks to be performed and issues to be discussed with the patient. The appointment includes discussion and guidance in various subjects related to diabetes care, such as diet, use of medication, foot care and physical activity. The nurses are also required to verify that all recommended laboratory tests and referrals have been performed.

The main outcome measures in this study were the following parameters of diabetes follow up: BP measurement, LDL, urine microalbumin, HbA1C and fundoscopy (each of them at least once a year). Complete diabetes follow up was defined as performing all of these tests at least once during a calendar year. We also compared control of BP, LDL and HbA1c levels between subject groups.

Data were retrieved regarding patients’ demographic, other chronic diseases, lab results, number of family physician visits and hospitalizations per calendar year. Patients with low socio-economic status (SES) were defined as those exempt from payments based on their income by the national insurance.

### Statistics

The sample size needed to meet the study objectives was initially calculated to be 508 patients. This would enable detection of a 1 mg % difference in HbA1c, assuming an SD of 3 and assuming that 75% of patients were seen by the nurse, with a power of 0.9 and a p value of 0.05. Since there were no strict guidelines controlling patient referral to the nurse and no previous data to rely on, we chose to sample four times this value.

Chi square and *t*-test were used for comparison of each variant. Logistic regression models were built to account for possible confounding, comparing the physician follow-up group to the intervention group for complete diabetic follow-up, achieving HbA1C goal (<7%), LDL goal (<100 mg %) and systolic BP goal (<130).

Note that Clalit Health Service has a free choice of primary care physician. Each patient can choose his/her own primary care physician and his/her primary care clinic. During the follow-up years patients could change the clinic, their family physician or the primary care nurse, so we were unable to control for the hierarchical nature of the data (patients nested within physicians and physicians nested within clinics).

The analysis was done for each year separately and for all three years together. STATA 8.0 statistical software (Stata Corp. College Station, TX, USA) was used for statistical analysis.

## Results

Of the 2,024 patients selected, 1,044 (51.6%) were female. The average age was 60.7 years (range 40–76) and 760 (37.5%) were from low SES. Table 1 demonstrates the patient characteristics for each of the study years.

**Table 1:** Patient characteristics

	**Physician only follow-up**	**Supplemental nursing follow-up**	**P-value**
**2005**	**1,223**	**801**	
Age (years, mean ± SD)	59.8 ± 9.0	62.1 ± 8.3	<0.001
Gender (% men)	47.3%	50.1%	0.231
Low SES (%)	33.5%	43.6%	<0.001
Immigrant (%)	40.1%	31.5%	<0.001
BMI (Kg/m^2^,mean ± SD)	29.7 ± 5.2	30.1 ± 5.6	0.092
At least one additional chronic disease (%)	78.6%	83.7%	<0.001
**2006**	**1,172**	**852**	
Age (years, mean ± SD)	59.8 ± 8.8	62.0 ± 8.6	<0.001
Gender (%men)	48.1%	48.8%	0.755
Low SES (%)	33.0%	43.8%	<0.001
Immigrant (%)	38.6%	33.9%	0.029
BMI (Kg/m^2^mean ± SD)	29.8 ± 5.3	30.0 ± 5.4	0.350
At least one additional chronic disease (%)	78.8%	83.1%	0.001
**2007**	**1,019**	**1,005**	
Age (years, mean ± SD)	59.2 ± 8.6	62.2 ± 8.7	<0.001
Gender (% men)	47.4%	49.4%	0.356
Low SES (%)	33.2%	42.0%	<0.001
Immigrant (%)	41.4%	31.8%	<0.001
BMI (Kg/m^2^mean ± SD)	30.0 ± 5.5	29.8 ± 5.2	0.508
At least one additional chronic disease (%)	78.6%	82.6%	<0.001
**Complete 3 years follow-up**	**1,616 (79.8%)**	**408 (20.2%)**	
Age (years, mean ± SD)	60.0 ± 8.8	63.6 ± 8.2	<0.001
Gender (% men)	52.6%	49.3%	0.295
Low SES (%)	35.0%	47.8%	<0.001
Immigrant (%)	38.7	28.6%	<0.001
BMI BMI (Kg/m^2^mean ± SD)	29.8 ± 5.4	30.1 ± 5.3	0.419
At least one additional chronic disease (%)	78.1%	86.3%	<0.001

In 2005, 801 diabetic patients (39.5% of the 2,024 study participants) received structured nursing follow-up for diabetes. In 2006, this value rose to 852 patients (42.1%) and in 2007 1,005 patients (49.6%) were enrolled in structured nursing follow-up care.

408 (20.1%) patients received structured nursing follow-up through all three study years. Patients receiving structured nursing follow-up care, tended to be older, and a higher proportion of these patients had a low SES and were also diagnosed with additional chronic diseases.

Health resource utilization was higher among patients receiving structured nursing follow-up. Their hospitalization rate was higher (13.7% vs. 10.5% in 2005 p = 0.030, 15.8% vs. 12.1% in 2006 p = 0.016, and 16.8% vs. 11.2% in 2007 p < 0.001). However, these differences disappeared when the results were adjusted for patients’ characteristics (age, gender, SES, immigrants, BMI, no. of chronic diseases). Physician visits (Table 2) were higher among the study group, and further adjustment for patients’ characteristics did not influence these values.

**Table 2:** Number of family physician’s visits per year (average ± SD)*

	**Physician only follow-up**	**Supplemental nursing follow-up**	**P-value**
2005	3.8 ± 3.8	6.8 ± 4.6	<0.001
2006	4.3 ± 3.9	7.3 ± 4.9	<0.001
2007	4.9 ± 3.8	7.2 ± 5.0	<0.001
Complete 3 years follow-up average visits per year	5.0 ± 3.4	7.7 ± 4.5	<0.001

*The differences did not change when adjusted for patients characteristics (age, gender, SES, immigrants, BMI, no. of chronic diseases).

Performance of complete diabetic follow-up is reported in Table 3. In each of the study years, complete diabetes follow-up protocol was more likely in the intervention group (2005: 21.5% vs 52.8%, 2006: 30.3% vs 55%, 2007 35.7% vs 52.5%). Similarly, patients in the nursing follow-up group were more likely to have complete diabetic follow-up in all three years (26% vs 10.4%).

**Table 3:** Performance of complete diabetes follow-up* (% of patients)

**Year**	**Physician only follow-up**	**Supplemental nursing follow-up**	**P-value**
2005	21.5%	52.8%	<0.001
2006	30.3%	55.5%	<0.001
2007	35.7%	52.5%	<0.001
Complete 3 years follow-up Complete diabetes follow- up in all 3 years	10.4%	26.0%	<0.001

*Complete diabetes follow-up was defined as performing of blood pressure measurement, LDL, microalbumin, and HbA1C tests and fundoscopy at least once during calendric year.

When potential confounders were taken into account, although the odds for complete follow-up were lower, they still remained over 1.4 times more likely in each of the follow-up years, for those receiving the nursing follow-up protocol (Table 4).

**Table 4:** Odds ratios of achieving treatment targets comparing nursing follow-up to physician follow-up only

	**OR unadjusted ± SD**	**p-value**	**OR model 1 ± SD**	**p-value**	**OR model 2 ± SD**	**p-value**	**OR model 3 ± SD**	**p-value**
**2005**								
Complete follow-up	4.08 ± 0.40	<0.001	3.87 ± 0.39	<0.001	1.97 ± 0.10	<0.001	1.96 ± 0.10	<0.001
HbA1c	0.86 ± 0.09	0.160	0.85 ± 0.08	0.097	0.89 ± 0.09	0.266	0.91 ± 0.10	0.383
LDL	1.44 ± 0.14	<0.001	1.41 ± 0.14	0.001	1.39 ± 0.14	0.001	1.34 ± 0.14	0.006
Systolic BP	0.71 ± 0.07	0.001	0.73 ± 0.07	0.004	0.74 ± 0.08	0.005	0.74 ± 0.08	0.007
**2006**								
Complete follow-up	2.87 ± 0.27	<0.001	2.77 ± 0.26	<0.001	2.10 ± 0.21	<0.001	2.13 ± 0.21	<0.001
HbA1c	0.83 ± 0.08	0.062	0.79 ± 0.08	0.017	0.83 ± 0.08	0.060	0.83 ± 0.08	0.072
LDL	1.19 ± 0.11	0.070	1.15 ± 0.11	0.150	1.14 ± 0.11	0.169	1.08 ± 0.11	0.431
Systolic BP	0.64 ± 0.06	<0.001	0.68 ± 0.07	<0.001	0.68 ± 0.07	<0.001	0.67 ± 0.07	<0.001
**2007**								
Complete follow-up	1.99 ± 0.18	<0.001	1.83 ± 0.17	<0.001	1.41 ± 0.07	<0.001	1.43 ± 0.07	<0.001
HbA1c	1.00 ± 0.09	0.969	0.91 ± 0.09	0.333	0.95 ± 0.09	0.647	0.95 ± 0.10	0.638
LDL	0.19 ± 0.11	0.069	1.09 ± 0.10	0.388	1.07 ± 0.10	0.4396	1.04 ± 0.10	0.705
Systolic BP	0.79 ± 0.07	0.001	0.79 ± 0.07	0.015	0.79 ± 0.07	0.014	0.79 ± 0.08	0.020
**3 years**								
Complete follow-up	3.02 ± 0.42	<0.001	2.60 ± 0.37	<0.001	2.22 ± 0.32	<0.001	2.04 ± 0.31	<0.001

Model 1 adjusted for age.

Model 2 adjusted for age and number of physician visits.

Model 3 adjusted for age, number of physician visits, low SES, chronic diseases, gender and immigrant status.

Minor differences between the groups were observed for systolic BP and HbA1C levels. More patients in the control group had controlled systolic BP. However, in the first two years of the study LDL levels were significantly lower in the nurse follow-up group (2005 110.0 vs 103.7 p < 0.001, 2006 110.8 vs 105.7 p < 0.001) (Table 5). When odds ratios were calculated for the likelihood of achieving treatment goals for LDL only, in the first year of the study, they improved in the nurse follow-up group. However, the improvement in 2006 was nullified after adjusting for potential confounding factors (Table 4).

**Table 5:** Follow-up results 2005-2007

**Year**	**Physician only follow-up (mean ± SD)**	**Supplemental nursing follow-up (mean ± SD)**	**P-value**
**HbA1C levels (%)**			
2005	7.5 ± 1.5	7.6 ± 1.5	0.057
2006	7.3 ± 1.3	7.5 ± 1.5	<0.001
2007	7.2 ± 1.3	7.2 ± 1.4	0.402
Complete 3 years follow-up results in 2007	7.2 ± 1.3	7.2 ± 1.4	0.692
**LDL cholesterol (mg %) levels**			
2005	110.0 ± 30.3	103.7 ± 29.7	<0.001
2006	110.8 ± 30.9	105.7 ± 28.3	<0.001
2007	99.9 ± 30.9	98.6 ± 28.9	0.371
Complete 3 years follow-up results in 2007	100.5 ± 30.4	94.2 ± 27.2	0.0003
**Systolic blood pressure (mm Hg)**			
2005	136.6 ± 16.4	137.0 ± 15.4	0.617
2006	134.8 ± 14.7	136.5 ± 14.5	0.020
2007	133.8 ± 14.8	133.8 ± 13.7	0.933
Complete 3 years follow-up results in 2007	133.8 ± 14.5	133.9 ± 13.2	0.816

## Discussion

The follow-up and treatment of diabetic patients is complex and requires cooperation between both patient and health professionals, and between different health professionals, to achieve optimal control. From a system-wide perspective, the efforts required to achieve good control among diabetic patients require vast resources from health care systems that are increasingly trying to improve their cost effectiveness.

In our study patients who were provided supplementary structured nursing follow-up in addition to the standard medical care were more likely to achieve complete diabetes maintenance care. There was no difference in systolic blood pressure levels between the groups, and no significant difference in HbA1c levels was observed. LDL targets were reached more frequently among those receiving supplementary follow-up in the first year of the study. However this effect did not carry through the three years of follow-up.

Other studies have demonstrated that nurse led interventions can improve cardiac risk factors in high-risk patients [9] and that nurse case managers using a treatment algorithm can improve the number of individuals with control of multiple cardiovascular risk factors [5]. Practice-based nurses accomplished comparable results with GPs regarding clinical parameters, and achieved better patient satisfaction [10] both at the one year of follow-up assessment. It is possible that due to the differences between the groups, that the clinical effects in our study were less significant than the effectiveness of the protocol on the process of diabetes management. It is also possible that, as was concluded by a Chochrane review, the clinical effects of nurse based interventions are only evident for up to 12 months [6].

Patients receiving supplemental nursing follow-up visited their family physician more frequently than did patients in the control group. It is reasonable to assume that this is a marker for the extensive work needed in order to achieve better diabetes control for these patients. It has been found that shorter encounter intervals were associated with faster decrease in blood pressure and earlier blood pressure normalization in diabetic patients [10]. Additionally, frequent primary care provider encounters were associated with fastest achievement of HbA1c, BP, and LDL cholesterol targets for diabetic patients [11].

### Limitations

The fact that patient allocation to the two groups was not random and that they were dissimilar in many respects, is a major limitation of this study. Patients in the supplementary follow-up group were older and a higher proportion of these patients had additional chronic diseases and they were more likely to be of a lower socioeconomic status. This is probably a reflection of the medical priorities implemented for patient selection. This selection bias may have hidden additional benefits in the nurse follow-up program. On the other hand, this process may also have led to the selection of patients who are more amenable for intervention [12]. Another limitation of the study is the fact that we could not account for clustering due to the manner in which we collected the data.

Multidisciplinary primary care teams have been found to be effective in improving diabetes control among patients [13]. This study represents the transition of an intervention program into daily routine work. In real life, “nurse time” is a limited resource and should be reserved for those patients who will benefit most from it.

Although the patients receiving supplemental nursing follow-up were older, had a higher incidence of chronic diseases as well as being from a poorer socio-economic background, they achieved better measures of process than the control group and had similar outcome results.

## Conclusion

This study indicates an added value to diabetic structured nursing follow-up in improving the process of diabetic care and possibly clinical control in more complex patients.

The results reinforce the importance of targeting professional resources and of the importance of teamwork, between the physicians and nurses in helping diabetic patients maximize the control of their diabetes. Further investigation is required to assess the effectiveness of nurse led programs to increase their impact on targeted patient groups.

## Competing interests

The authors declare that they have no competing interests.

## Authors’ contributions

All authors have made substantial contributions to conception and design, involved in drafting the manuscript, read and approved the final manuscript. MS has substantial contribution to data analysis.

## Authors’ information

Michal Shani MD MPH is a family physician at the Department of Family Medicine Central District, Clalit Health Service, and at the Department of Family Medicine Sackler Faculty of Medicine, Tel Aviv University, Israel.

Sasson Nakar MD, was a family physician and head of the Department of Family Medicine for the Central District of Clalit Health Service. He passed away in May 2013.

Alex Lustman MD MPH is a family physician at the Department of Family Medicine Central District, Clalit Health Service, and at the Department of Family Medicine Sackler Faculty of Medicine, Tel Aviv University, Israel

Amnon Lahad MD MPH is a family physician, and head of the Family Medicine Department of the Hadassah Medical School, The Hebrew University, Israel.

Shlomo VInker MD MHA is a family physician at the Department of Family Medicine Central District, Clalit Health Service, and at the Department of Family Medicine Sackler Faculty of Medicine, Tel Aviv University, Israel.
